# Lactate as an Astroglial Signal Augmenting Aerobic Glycolysis and Lipid Metabolism

**DOI:** 10.3389/fphys.2021.735532

**Published:** 2021-09-30

**Authors:** Anemari Horvat, Robert Zorec, Nina Vardjan

**Affiliations:** ^1^Laboratory of Neuroendocrinology – Molecular Cell Physiology, Institute of Pathophysiology, Faculty of Medicine, University of Ljubljana, Ljubljana, Slovenia; ^2^Laboratory of Cell Engineering, Celica Biomedical, Ljubljana, Slovenia

**Keywords:** L-lactate, L-lactate sensitive receptors, astrocytes, cAMP, aerobic glycolysis, lipid metabolism

## Abstract

Astrocytes, heterogeneous neuroglial cells, contribute to metabolic homeostasis in the brain by providing energy substrates to neurons. In contrast to predominantly oxidative neurons, astrocytes are considered primarily as glycolytic cells. They take up glucose from the circulation and in the process of aerobic glycolysis (despite the normal oxygen levels) produce L-lactate, which is then released into the extracellular space *via* lactate transporters and possibly channels. Astroglial L-lactate can enter neurons, where it is used as a metabolic substrate, or exit the brain *via* the circulation. Recently, L-lactate has also been considered to be a signaling molecule in the brain, but the mechanisms of L-lactate signaling and how it contributes to the brain function remain to be fully elucidated. Here, we provide an overview of L-lactate signaling mechanisms in the brain and present novel insights into the mechanisms of L-lactate signaling *via* G-protein coupled receptors (GPCRs) with the focus on astrocytes. We discuss how increased extracellular L-lactate upregulates cAMP production in astrocytes, most likely *via*L-lactate-sensitive G_s_-protein coupled GPCRs. This activates aerobic glycolysis, enhancing L-lactate production and accumulation of lipid droplets, suggesting that L-lactate augments its own production in astrocytes (i.e., metabolic excitability) to provide more L-lactate for neurons and that astrocytes in conditions of increased extracellular L-lactate switch to lipid metabolism.

## Introduction

Historically, L-lactate was first considered as a cellular waste product of glycolytic metabolism, however, it was later proposed that L-lactate can also act as a supplemental oxidative energy substrate and as a signaling molecule in the brain (Dienel, 2012a; Magistretti and Allaman, 2018).

L-Lactate is involved in various cellular processes in the brain, including in the regulation of intracellular Ca^2+^ signaling (Requardt et al., 2012), cell energy metabolism (Bergersen and Gjedde, 2012; Barros, 2013), activity of various channels and transporters (Gordon et al., 2008; Ohbuchi et al., 2010), myelination (Fünfschilling et al., 2012), and gene expression (Yang et al., 2014; Descalzi et al., 2019). L-Lactate was shown to support high-level cognitive functions, learning and long-term memory formation (Newman et al., 2011; Suzuki et al., 2011; Gibbs, 2015; Murphy-Royal et al., 2020), and may have a neuroprotective role against excitotoxicity (Ros et al., 2001) and ischemia (Berthet et al., 2012; Castillo et al., 2015). Moreover, impaired L-lactate signaling and metabolism have been associated with several brain pathologies, such as epilepsy (Yang et al., 2016), depression (Carrard et al., 2018), and neurodevelopmental disorders [e.g., X-linked intellectual disability (XLID); D’Adamo et al., 2021]. However, the molecular mechanisms of the broad spectrum of L-lactate functions in the brain in health and disease are not yet clear (Dienel, 2012a) and may involve the role of L-lactate as an energy substrate and as a signaling molecule in the brain.

The brain is composed of various cell types with distinct metabolic profiles. Under physiologic conditions, astrocytes and mature oligodendrocytes are considered mainly glycolytic cells, whereas neurons and microglia are predominantly oxidative (Fünfschilling et al., 2012; Zhang et al., 2014; Sharma et al., 2015; Afridi et al., 2020; Yang et al., 2021). A high glycolytic rate with L-lactate production is present in astrocytes and oligodendrocytes during increased neuronal activity despite normal brain oxygen levels, a process termed aerobic glycolysis (Pellerin and Magistretti, 1994; Barros, 2013; Saab et al., 2016), also known as the Warburg effect initially described in fast proliferating cancer cells (Warburg, 1956; Vander Heiden et al., 2009). To avoid L-lactate-mediated intracellular acidification, which causes negative feedback on glycolytic flux (Hertz et al., 2014), L-lactate is shuttled intra-/intercellularly and extracellularly by diffusion down its concentration gradient *via* membrane monocarboxylate transporters (MCTs; Pérez-Escuredo et al., 2016), K^+^/voltage-sensitive cation channels (Sotelo-Hitschfeld et al., 2015), pannexin and connexin hemichannels (Karagiannis et al., 2016), and gap junctions (Scemes et al., 2017). Shuttling of brain L-lactate enables neurons to accept L-lactate and use it as an energy fuel in oxidative metabolism (Hertz et al., 2014). L-Lactate can also act as a signaling molecule intracellularly or extracellularly indirectly or *via* receptor-mediated signaling mechanisms (Barros, 2013; Mosienko et al., 2015). Due to its signaling characteristics and shuttling capability, L-lactate may act as a brain “volume transmitter”, activating L-lactate-sensitive receptors (LLRs) on neural cells that are relatively distant from the site of L-lactate release. In this way, L-lactate-mediated signals could spread over larger areas of the brain (Bergersen and Gjedde, 2012).

In this review, we first discuss the current knowledge on intracellular and extracellular L-lactate signaling mechanisms in the brain with an emphasis on receptor-based signaling in astrocytes, where L-lactate is predominantly produced in the brain. Then, we discuss how extracellular L-lactate augments astroglial aerobic glycolysis and thus its own production and how this may contribute to L-lactate “volume transmission”. The role of L-lactate as a metabolic substrate and signal in the control of brain lipid metabolism is also addressed.

## Molecular Mechanisms of L-Lactate Signaling in the Brain

In the brain, L-lactate can exert its role as a signaling molecule through several intracellular and extracellular mechanisms.

### Intracellular L-Lactate Signaling in the Brain

Once inside the cells, L-lactate can modulate brain function indirectly by changing the intracellular redox state of cells as glycolytic transformation of L-lactate into pyruvate generates NADH and thus increases the NADH/NAD^+^ ratio (Hung et al., 2011; Hertz et al., 2014). By altering the cellular redox state, L-lactate (1) promotes the expression of synaptic plasticity-related genes, such as Arc, c-Fos, and Zif268, by potentiating ionotropic glutamate receptor (NMDA receptor)-mediated Ca^2+^ currents induced by glutamate and glycine leading to activation of a downstream Erk1/2 signaling cascade in neurons *in vitro* and *in vivo* (Yang et al., 2014) and (2) modulates astroglial Ca^2+^ signaling by increasing the frequency of dopamine-induced Ca^2+^ signals (Requardt et al., 2012). The entry of L-lactate into cells can also affect the cell energy status where L-lactate is first metabolized to pyruvate, which is then used for generation of ATP in the tricarboxylic acid cycle (TCA) leading to an increased ATP/ADP ratio. This was shown to regulate the activity of ATP-sensitive K^+^ channels in hypothalamic and orexin neurons, which close when cytoplasmic ATP levels increase, leading to depolarization of the membrane (Song and Routh, 2005; Parsons and Hirasawa, 2010; Mosienko et al., 2015). Lastly, L-lactate uptake *via* MCTs is accompanied by the cotransport of protons, causing intracellular acidification (Nedergaard and Goldman, 1993), which can modulate brain energy metabolism by inhibiting phosphofructokinase (PFK), a glycolytic enzyme extremely sensitive to small changes in pH (Dienel, 2012b), and potentially other nearby ion channels, transporters, and receptors.

### Extracellular L-Lactate Signaling in the Brain

Some actions of L-lactate cannot be attributed to its intracellular signaling activity, but can only be explained by L-lactate acting extracellularly as a signaling molecule. Increases in extracellular L-lactate levels that occur in the brain in response to (1) increased brain activity, (2) low oxygen availability, both triggering glycolysis and L-lactate release from neural cells, and/or (3) increased blood L-lactate levels (Boumezbeur et al., 2010; Mosienko et al., 2015) were linked to various cellular responses in the brain. Increased extracellular L-lactate due to low oxygen levels in the brain hinders prostaglandin E_2_ (PGE_2_) clearance, a known vasodilator, from the extracellular space by affecting prostaglandin transporter efficacy. Consequently, PGE2 concentration in the extracellular space increases, resulting in vasodilation (Gordon et al., 2008). Moreover, in rat hypothalamic vasopressin neurons, extracellular L-lactate was shown to potentiate the activity of acid-sensing ion channels (ASICs), voltage-insensitive cationic channels activated by extracellular acidification. In these neurons, L-lactate (15mM), through chelation of extracellular Ca^2+^, which competes with H^+^ at the activation site of ASICs, increases the sensitivity of ASICs to H^+^ leading to enhanced acid-induced currents (Ohbuchi et al., 2010). Various recent studies suggest that extracellular L-lactate can also activate LLRs on neural cells (Table 1), causing activation of glucose and lipid metabolism in primary cortical astrocytes, modulation of neuronal activity of primary cortical neurons, and release of noradrenaline from noradrenergic neurons (Bozzo et al., 2013; Lauritzen et al., 2014; Tang et al., 2014; Mosienko et al., 2018; Vardjan et al., 2018; D’Adamo et al., 2021).

**Table 1:** Astroglial and neuronal L-lactate-sensitive receptors and their effects on intracellular signaling and metabolism.

Receptor	G-protein coupling	L-Lactate sensitivity	Intracellular signaling		Metabolic effects		Other agonists	References
			Astrocytes	Neurons	Astrocytes	Neurons		
GPR81 (HCAR1)	G_i_	~4–30mM	n.d.	↓Ca^2+^-transient frequency	n.d.	n.d.	3,5-DHBA α-HBA Glycolate γ-HBA 3Cl-5OH-BA Compound 2	Liu et al., 2009, 2012; Dvorak et al., 2012; Bozzo et al., 2013; Lauritzen et al., 2014; Sakurai et al., 2014
Neuronal LLRx	G_s_	0.5mM	n.d.	↑[cAMP]_i_ ↑PKA activity	n.d.	n.d.	D-Lactate (antagonist) MPA aHIBA HMBA 2HPA KA	Tang et al., 2014; Mosienko et al., 2018
Astroglial unidentified LLR	G_s_	20mM	↑[cAMP]_i_ ↑PKA activity	n.d.	↓[glucose]_i_ ↑[lactate]_i_ ↑lipid droplet accumulation	n.d.	3Cl-5OH-BA Compound 2	Vardjan et al., 2018; D’Adamo et al., 2021; Smolič et al., 2021
Olfr78^*^ (OR51E2)	G_s_	~4mM	/	n.d.	/	n.d.	Acetate Propionate	Conzelmann et al., 2000; Chang et al., 2015; Mosienko et al., 2018
GPR4	Presumable allosteric modulation	1–10mM	n.d.	n.d.	n.d.	n.d.	H+	Hosford et al., 2018

GPR81, G-protein coupled receptor 81; HCAR1, hydroxycarboxylic acid receptor 1; LLR, L-lactate-sensitive receptor; Olfr78, L-lactate-sensitive olfactory receptor 78; OR51E2, human orthologue of L-lactate-sensitive olfactory receptor 78; GPR4, proton-sensitive G-protein coupled receptor 4; 3,5-DHBA, 3,5-dihydroxybenzoic acid; α-HBA, α-hydroxybutyrate; γ-HBA, γ-hydroxybutyrate; 3Cl-5OH-BA, 3-chloro-5-hydroxybenzoic acid; Compound 2, 2,4-methyl-N-(5-(2-(4-methylpiperazin-1-yl)-2-oxoethyl)-4-(2-thienyl)-1,3-thiazol-2-yl) cyclohexanecarboxamide; MPA, (S)-(–)-2-methoxypropionic acid; aHIBA, α-hydroxyisobutyric acid; HMBA, 2-hydroxy-3-methyl-butyric acid; 2HPA, (S)-2-hydroxypentanoic acid; KA, kynurenic acid; PKA, protein kinase A; [cAMP]_i_, intracellular concentration of cAMP; [glucose]_i_, intracellular concentration of free D-glucose; [lactate]_i_, intracellular concentration of L-lactate; and n.d., not determined.

* Not expressed in astrocytes.

L-Lactate is a weak agonist of the G_i/o_-protein coupled hydroxycarboxylic acid receptor 1 (HCAR1 or HCA1; EC_50_ of 1–5mM; Liu et al., 2009), formerly known as orphan G-protein coupled receptor 81 (GPR81). GPR81 was first discovered in adipose tissue (Cai et al., 2008; Liu et al., 2009) and later researched in various cancers and cancer cell lines (Baltazar et al., 2020) and skeletal muscle (Rooney and Trayhurn, 2011). Although the expression level of GPR81 in the brain cells appears to be negligible according to the RNA sequencing databases and proteomic analysis (Zhang et al., 2014; Sharma et al., 2015; Zhang et al., 2016), GPR81 has been detected in the brain tissue by anti-GPR81 antibodies (Lauritzen et al., 2014). According to this study, cerebral GPR81 is concentrated predominantly in the postsynaptic membranes of the excitatory neuronal synapses, but can also be found, although to a much lesser extent, at the perisynaptic astroglial processes and at the blood-brain barrier, in particular in endothelial cells and perivascular astrocytic processes (Lauritzen et al., 2014). Consistent with the studies using anti-GPR81 antibodies, quantitative RT-PCR experiments confirmed the expression of GPR81 in mouse brain, including the cerebellum, hippocampus, and cerebral cortex (Lauritzen et al., 2014), and in isolated rat and mouse cortical astrocytes (Vardjan et al., 2018). Although under physiologic conditions, extracellular concentrations of L-lactate, that are fluctuating between the sub- and low millimolar range (0.1–1.4mM) in rodent brain and around 5mM in human brain, as measured by microdialysis in different brain areas (Abi-Saab et al., 2002; Mosienko et al., 2015), might be too low to fully activate GPR81, brain extracellular L-lactate concentrations can increase to several millimolar (Mosienko et al., 2015) under certain (patho)physiologic conditions, which could activate GPR81. For instance, (1) during exercise, when L-lactate blood levels increase and L-lactate enters the brain from the systemic circulation (usually the L-lactate concentration in the brain is lower than in the circulation; Bergersen, 2015), as GPR81-mediated effects of exercise on brain function were demonstrated in mice that have been subjected to high-intensity interval exercise or L-lactate injection mimicking exercise-induced increase in blood L-lactate levels (Morland et al., 2017); (2) when oxygen or glucose supplies in the brain are low (e.g., during hypoxia, ischemia, seizures, and hyperglycemia; Smith et al., 1986; During et al., 1994; Lee et al., 2015; Mosienko et al., 2015); and (3) when the gene expression profile of neural cells is changed favoring L-lactate production/accumulation (Afridi et al., 2020). Moreover, one can speculate that under physiologic conditions in response to increased neuronal activity, L-lactate is released from neural cells locally, in microdomains. In microdomains, the L-lactate concentration is likely high enough to fully activate GPR81, however, the presence of such microdomains in the brain needs to be determined in the future (Morland et al., 2015; Mosienko et al., 2015).

Activation of the GPR81 in adipose tissue through G_i/o_-proteins downregulates the formation of cAMP. This leads to the inhibition of lipolysis, promoting lipid storage in adipocytes (Ahmed et al., 2010). Similar to adipocytes, GPR81 activation in cancer and muscle cells decreases cAMP levels (Sun et al., 2016; Feng et al., 2017), which is crucial for cancer cell survival (Roland et al., 2014) and maintenance of mitochondrial function (Sun et al., 2016; Baltazar et al., 2020), respectively. Consistent with these results, in rat hippocampal slices, increase in forskolin-induced cAMP was inhibited by L-lactate at concentrations >10mM and by selective GPR81 receptor agonist 3,5-dihydroxybenzoic acid (3,5-DHBA) with half maximal inhibitory concentration (IC_50_) of 1.4mM, as measured by cAMP radioimmunoassay on homogenized brain slices (Lauritzen et al., 2014). Moreover, L-lactate (in a concentration-dependent manner with IC_50_ of ~4.2mM) and selective GPR81 agonist 3,5-DHBA (1mM) decreased the spontaneous electrical activity of isolated mouse cortical neurons measured as a decrease in Ca^2+^-transient frequency. This most likely occurs *via* G_i_-protein activation given that pertussis toxin, an inhibitor of G_i_-proteins, prevented the decrease of neuronal activity by L-lactate (Bozzo et al., 2013), suggesting that brain GPR81 is also coupled to G_i/o_-proteins and responds only to supraphysiologic L-lactate concentrations (Lauritzen et al., 2014).

Recently, it has been proposed that noradrenergic neurons (Tang et al., 2014; Mosienko et al., 2018) and cortical astrocytes (Vardjan et al., 2018; D’Adamo et al., 2021) may respond to extracellular L-lactate through as yet unidentified LLRs that are coupled to G_s_-proteins and cAMP production, which is discussed in more detail in the following section.

## Receptor-Mediated L-Lactate Signaling in Astrocytes

Astrocytes, although electrically silent cells, can respond to many, if not all, signaling molecules in the brain (e.g., glutamate, ATP, noradrenaline, GABA, acetylcholine, serotonin, dopamine, cannabinoid, and bradykinin) through metabotropic G-protein coupled receptors (GPCRs) expressed on their surface. Activation of astroglial GPCRs can change intracellular Ca^2+^ and/or cAMP signals (i.e., cytoplasmic excitability) *via* receptor coupling to G_q_- and/or G_s_- and G_i/o_-proteins, respectively (Vardjan and Zorec, 2015), which affects astrocyte function and control of brain homeostasis (Verkhratsky and Nedergaard, 2018). Recently, extracellular L-lactate was identified as a novel signaling molecule in the brain that could excite L-lactate-sensitive GPCRs. Initially, GPR81 receptor, coupled to G_i/o_-proteins and downregulation of cAMP production, was suggested to be involved in L-lactate signaling in astrocytes (Lauritzen et al., 2014), but recently, another as yet unidentified GPCR, most likely coupled to G_s_-proteins and upregulation of cAMP production, has been linked to L-lactate-mediated signaling in astrocytes (Vardjan et al., 2018; D’Adamo et al., 2021).

**Figure 1:**
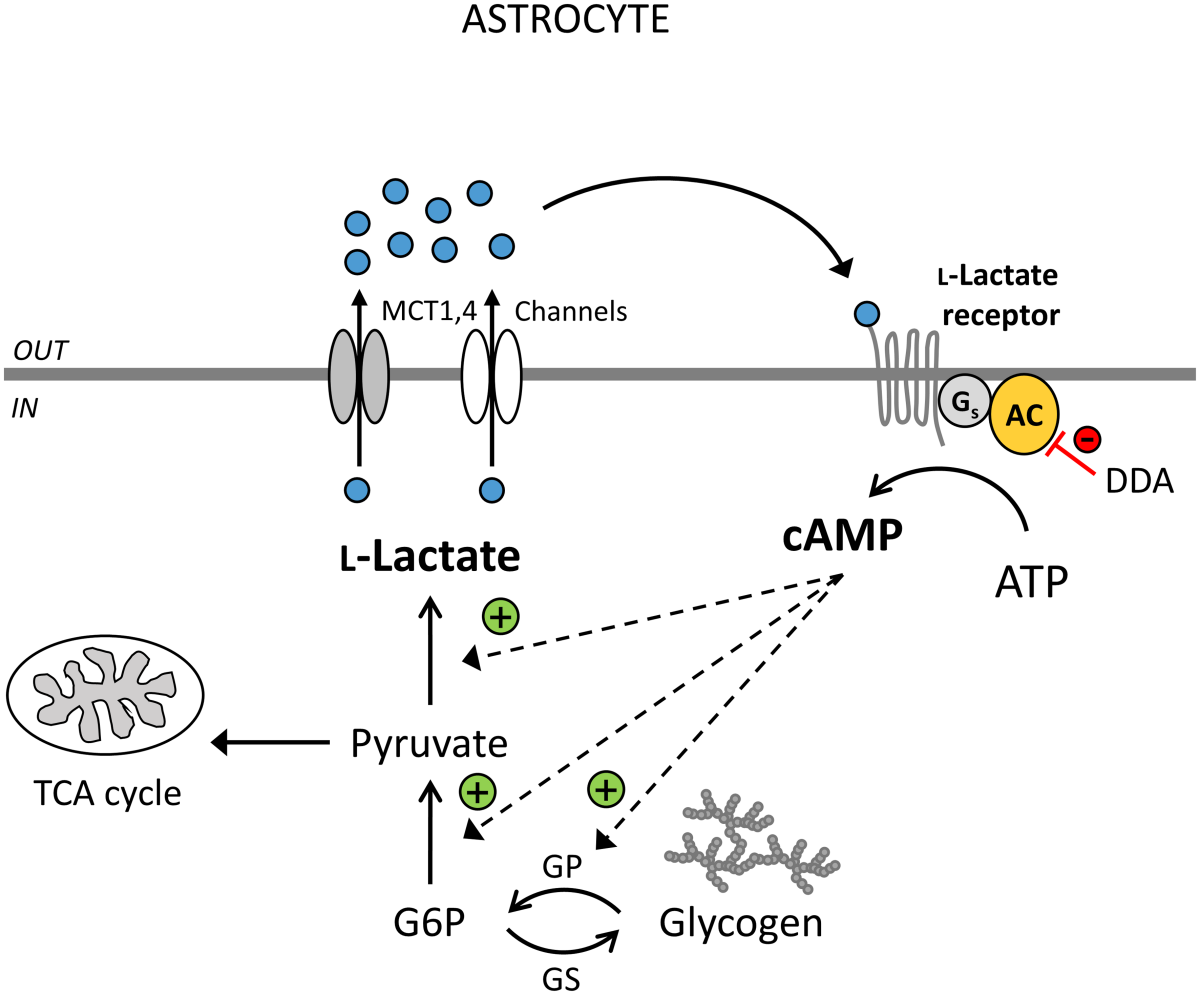
Extracellular L-lactate enhances cytosolic L-lactate production *via* yet unidentified receptors coupled to adenylate cyclase (AC) activity and cAMP signaling in astrocytes. L-Lactate (blue circles) is formed in astrocytes (IN) in the process of aerobic glycolysis and released through monocarboxylate transporters (MCTs) 1,4 and/or L-lactate-permeable channels. Extracellularly (OUT), L-lactate can be transported to neighboring cells as a fuel, it can exit the brain *via* the circulation, or act as a signaling molecule. By binding to the L-lactate-sensitive receptors (LLRs) on the surface of astrocytes, it can stimulate AC and cAMP production. This triggers glycogen degradation, glycolysis, and more L-lactate production. The inhibition of AC by 2',5'-dideoxyadenosine (DDA) causes a reduction in the astroglial LLR-mediated increase in cAMP and L-lactate levels (red line). L-Lactate-positive feedback mechanism (“metabolic excitability”) in astrocytes may maintain the L-lactate tissue concentration gradient between astrocytes and neighboring cells, enhancing the availability of L-lactate as a metabolic fuel when brain energy demands are high. DDA, 2',5'-dideoxyadenosine, an inhibitor of AC; TCA cycle, tricarboxylic acid cycle; G6P, glucose 6-phosphate; GP, glycogen phosphorylase; and GS, glycogen synthase. Channels denote lactate-permeable K^+^/voltage-sensitive cation channels, pannexin and connexin hemichannels.

Intracellular Ca^2+^ and cAMP imaging of rat cortical astrocytes revealed that Ca^2+^ signals in astrocytes, preloaded with a Ca^2+^ indicator Fluo-4 AM (D’Adamo et al., 2021), are not affected by extracellular L-lactate (20mM) and a selective GPR81 agonist, 3-chloro-5-hydroxybenzoic acid (3Cl-5OH-BA; 0.5mM; Vardjan et al., 2018; D’Adamo et al., 2021), while both agonists trigger a persistent increase in intracellular cAMP and protein kinase A (PKA) activity in astrocytes. The latter occurs within ~100s (cAMP) and ~200s (PKA) upon stimulation, as measured by genetically encoded fluorescence resonance energy transfer (FRET)-based cAMP sensor Epac1-camps and a cAMP-dependent PKA activity sensor AKAR2 (Vardjan et al., 2018; D’Adamo et al., 2021). The L-lactate-induced increase in cAMP depends on the activity of transmembrane adenylate cyclase (AC; Figure 1), given that the treatment of cells with an AC inhibitor 2',5'-dideoxyadenosine (DDA; 100μM) reduced the 20mM L-lactate-induced increase in cAMP levels by ~50%, consistent with a G_s_-protein signaling mechanism. Surprisingly, 3Cl-5OH-BA (0.5mM) and a high-affinity GPR81 agonist, 2,4-methyl-N-(5-(2-(4-methylpiperazin-1-yl)-2-oxoethyl)-4-(2-thienyl)-1,3-thiazol-2-yl) cyclohexanecarboxamide (Compound 2; 50nM; Sakurai et al., 2014) also trigger increases in cAMP in cortical astrocytes isolated from GPR81 knockout (KO) mice (Vardjan et al., 2018), indicating that L-lactate-triggered cAMP increases in astrocytes are independent of GPR81 receptor activation (Vardjan et al., 2018). These data obtained by real-time fluorescence microscopy contrast with the results obtained on adult rat hippocampal slices, where downregulation of cAMP production upon stimulation of tissue with extracellular L-lactate and selective GPR81 agonist 3,5-DHBA was linked to the activation of brain GPR81. In hippocampal slices, L-lactate and GPR81 agonist 3,5-DHBA exhibited concentration-dependent inhibition of forskolin-stimulated cAMP production with IC_50_ of ∼29 mM and 1.4mM, respectively. However, the brain cell type responsible for the observed downregulation of cAMP signals in hippocampal slices was not identified, as cAMP content was determined on homogenized tissue samples containing all brain cells (Lauritzen et al., 2014), suggesting that brain cells other than astrocytes, which express only low amounts of GPR81 (Lauritzen et al., 2014; Zhang et al., 2014; Sharma et al., 2015; Zhang et al., 2016), are responsible for L-lactate-mediated downregulation of cAMP generation in hippocampal slices.

Thus, astrocytes, in addition to GPR81, most likely express another, yet unidentified LLR coupled to G_s_-proteins and AC-mediated cAMP production, which is also activated by GPR81 agonists and responds with increases in cAMP only to supraphysiologic (20mM) extracellular L-lactate concentrations (Table 1), given that 2mM extracellular L-lactate concentration did not affect intracellular cAMP signals in astrocytes (Vardjan et al., 2018; D’Adamo et al., 2021). Interestingly, astroglial sensitivity to L-lactate-mediated cAMP elevation was increased in *Gdi1* KO cortical astrocytes isolated from a mouse model of *GDI1*-associated XLID (D’Adamo et al., 2021), a form of neurodevelopmental disorder characterized by “pure” mental deficiency (Curie et al., 2009). *GDI1* encodes for αGDI (Rab GDP dissociation inhibitor alpha) protein which regulates the GDP/GTP exchange reaction of most Rab proteins that are associated with vesicle traffic of molecules between cellular organelles (Stenmark, 2009). Namely, in *Gdi1* KO astrocytes, but not *Gdi1* WT astrocytes, extracellular L-lactate triggered intracellular cAMP increases already at a physiologic L-lactate concentration of 2mM (D’Adamo et al., 2021), possibly due to altered expression level of the astroglial LLR and/or downstream signaling factors, which may contribute to the metabolic imbalance and disease in this form of neurodevelopmental disorder (D’Adamo et al., 2021).

Consistent with the results obtained on rat and mouse isolated cortical astrocytes, the existence of a neuronal LLR that activates AC and cAMP production, named LLRx, was proposed in *locus coeruleus* (LC) noradrenergic neurons, which can, in contrast to an astroglial LLR, respond to physiologic extracellular L-lactate concentrations (Table 1; Tang et al., 2014; Mosienko et al., 2018). Studies performed on brainstem organotypic-cultured slices from rat pups containing LC noradrenergic neurons showed that exogenously applied L-lactate (2mM), as well as L-lactate released from astrocytes in response to optogenetic excitation, trigger depolarizations in noradrenergic neurons and subsequent release of stress response neuromodulator noradrenaline. The latter was suppressed if the slices were treated with oxamate (20mM), an L-lactate synthesis inhibitor, or 1,4-dideoxy-1,4-imino-d-arabinitol (DAB; 500μM), a glycogen shunt activity inhibitor, implying that astroglial-derived L-lactate is involved in activation of noradrenergic neurons. Moreover, treatment of slices with AC inhibitor SQ22536 (100μM) and PKA inhibitor H89 (10μM) suppressed the depolarizing effect of 2mM L-lactate, indicating involvement of G_s_-protein coupled receptors and the cAMP/PKA signaling pathway in L-lactate-mediated activation of noradrenergic neurons. The authors also propose that noradrenaline released from noradrenergic neurons can then back-excite neighboring astrocytes (Tang et al., 2014; Mosienko et al., 2018), most likely *via* astroglial adrenergic receptors (Bekar et al., 2008; Hertz et al., 2010; O’Donnell et al., 2012; Ding et al., 2013; Vardjan et al., 2014; Horvat et al., 2016), which may affect astrocyte function, including glucose metabolism, which is highly regulated in the brain by the activity of noradrenergic neurons (Bélanger et al., 2011; O’Donnell et al., 2012; Gibbs, 2015; Dienel and Cruz, 2016; Vardjan and Zorec, 2017; Bak et al., 2018).

So far, two G_s_-coupled LLRs were identified in the brain: olfactory receptor Olfr78 (human ortholog OR51E2) in mouse olfactory sensory neurons in certain brain areas (i.e., brainstem and nucleus tractus solitarius; Conzelmann et al., 2000) and GPR4 expressed in neurons in various rodent brain areas, such as retrotrapezoid and raphe nuclei, rostral ventrolateral medulla, septum, and LC (Table 1; Mosienko et al., 2017; Hosford et al., 2018; Mosienko et al., 2018). According to the RNA sequencing database (Zhang et al., 2014), Olfr78 and GPR4 expression in astrocytes is negligible, and most likely does not contribute to the observed L-lactate-induced increases in cAMP signals in astrocytes.

## Extracellular L-Lactate and Control of Astroglial Aerobic Glycolysis

Astrocytes are key neural cells controlling metabolic homeostasis of the brain (Verkhratsky and Nedergaard, 2018). Due to their specific glycolytic profile, they are the main site of L-lactate production and release and an almost exclusive store of glycogen in the brain, which they can rapidly mobilize to enter the glycolytic pathway (Magistretti and Allaman, 2015; Oz et al., 2015; Bak et al., 2018).

As a response to increased neuronal activity, astrocytes upregulate glucose metabolism, i.e., glucose uptake from the circulation, glycogenolysis, and aerobic glycolysis with L-lactate production (Pellerin and Magistretti, 1994; Hertz et al., 2015; Dienel and Cruz, 2016). Astrocytes can sense neuronal activity *via* changes in extracellular K^+^ (Bittner et al., 2011; Sotelo-Hitschfeld et al., 2015), and glutamate levels (Pellerin and Magistretti, 1994), both tightly coupled with Na^+^ fluxes across the membrane (Bittner et al., 2011; Chatton et al., 2016; Rose and Verkhratsky, 2016). Astrocytes respond to local increase in extracellular K^+^ with plasma membrane depolarization leading to an increase in intracellular pH, mediated by an electrogenic Na^+^/HCO_3_^−^ cotransporter (NBCe1), which stimulates aerobic glycolysis (Ruminot et al., 2011), most likely through activation of PFK, a pH-sensitive glycolytic enzyme (Dienel, 2012b) and/or HCO_3_^−^-mediated activation of soluble AC (Choi et al., 2012). On the other hand, extracellular glutamate stimulates astroglial aerobic glycolysis to provide energy for the activity of the Na^+^/K^+^ ATPase pump, which is activated by an increase in the intracellular concentration of Na^+^ due to Na^+^-glutamate cotransport into astrocytes (Pellerin and Magistretti, 1994, 1996; Bittner et al., 2011). Active neurons also release NH_4_^+^, a by-product of catabolism, which in astrocytes causes an increase in intracellular L-lactate concentration *in vitro* and *in vivo*. However, the effect of NH_4_^+^ on L-lactate production is not due to glycolytic stimulation, instead it affects mitochondrial pyruvate shunting by diverting the flux of pyruvate from mitochondria to L-lactate production (Lerchundi et al., 2015). Moreover, astrocytes can also sense increased neuronal activity *via* signaling molecules released from activated neurons. The latter bind to astroglial metabotropic GPCRs and ionotropic receptors (Verkhratsky and Nedergaard, 2018), which leads to intracellular increases in Ca^2+^ and/or cAMP signals in astrocytes (Vardjan and Zorec, 2015). In astrocytes, both Ca^2+^ and cAMP can increase glycogenolysis and aerobic glycolysis with L-lactate production (Horvat et al., 2021). Astrocytes are also active participants in the neurovascular unit where they can respond to nitric oxide (NO) released by endothelial cells. NO inhibits astrocytic respiration and stimulates aerobic glycolysis, resulting in glucose depletion and L-lactate production *via* inhibition of mitochondrial cytochrome oxidase (Almeida et al., 2004; San Martín et al., 2017) and increased activity of 6-phosphofructo-1-kinase (PFK1), a master regulator of glycolysis (Almeida et al., 2004).

L-Lactate is not only produced but also released from astrocytes into the extracellular space *via* plasmalemmal lactate transporters MCT1 and 4, putative K^+^/voltage-sensitive cation channels (Sotelo-Hitschfeld et al., 2015), and/or hemichannels (Karagiannis et al., 2016). The presence of plasmalemmal L-lactate transporters in astrocytes and other brain cells represents the basis for a flux of L-lactate along its concentration gradient from astrocytes to (1) other brain cells, most importantly neurons (Pellerin and Magistretti, 1994, 2012) or (2) the circulation to exit the brain (Dienel, 2012a). According to the astrocyte-neuron L-lactate shuttle (ANLS) hypothesis, astroglial-derived extracellular L-lactate is taken up by neurons *via* the MCT2 transporters and fuels neuronal oxidative metabolism (Pellerin and Magistretti, 1994, 2012; Mächler et al., 2016), especially when energy demands are high, which is particularly important during memory formation and consolidation (Newman et al., 2011; Suzuki et al., 2011; Hertz et al., 2013; Descalzi et al., 2019; Murphy-Royal et al., 2020). Despite growing evidence supporting the ANLS hypothesis, the research community is not unanimous on this topic (Dienel, 2017, 2019). Some data challenge this hypothesis by showing that during brain activation neurons in acute mouse hippocampal brain slices and *in vivo* rely on their own L-lactate production rather than L-lactate derived from astrocytes to meet the increased energy demands, while ANLS may possibly function at rest, as studied by real-time two-photon fluorescence lifetime imaging microscopy (FLIM) of NADH dynamics (Díaz-García et al., 2017). One of the main arguments based on which the existence of an ANLS hypothesis has been questioned is that most of the past work supporting this process has been performed on primary astrocytes and neurons. However, recently ANLS was described by real-time two-photon FRET microscopy and L-lactate nanosensor also *in vivo* in mice (Mächler et al., 2016) and *Drosophila* (Liu et al., 2017) and was shown to be impaired in the *in vivo* mice models of Alzheimer’s disease (Sun et al., 2020).

Astroglial-derived extracellular L-lactate may also act extracellularly as a signaling molecule, activating LLRs on the surface of neural cells as discussed in the previous section (Table 1; Tang et al., 2014; Mosienko et al., 2015, 2018; Vardjan et al., 2018; D’Adamo et al., 2021). L-Lactate released from astrocytes was shown to excite noradrenergic neurons *via* AC-mediated cAMP signaling to release noradrenaline, which can then back-excite astrocytes (Tang et al., 2014). Activation of astroglial α_1_- and β-adrenergic receptors and intracellular Ca^2+^ and cAMP signals by noradrenaline is known to upregulate glucose uptake, glycogenolysis, and aerobic glycolysis in astrocytes, which can lead to more L-lactate production and release (Sorg and Magistretti, 1991; Gibbs, 2015; Horvat et al., 2017; Vardjan et al., 2018; Velebit et al., 2020; Fink et al., 2021). Moreover, extracellular L-lactate (20mM), as well as GPR81 agonist 3Cl-5OH-BA (0.5mM), were shown to upregulate aerobic glycolysis and L-lactate production in isolated cortical astrocytes as measured by Laconic, a FRET-based lactate nanosensor (Vardjan et al., 2018; D’Adamo et al., 2021). 3Cl-5OH-BA-induced L-lactate production in astrocytes was greatly reduced in the presence of AC inhibitor DDA (100μM), suggesting the involvement of a G_s_-protein coupled LLR and cAMP signals in the regulation of L-lactate-induced aerobic glycolysis in astrocytes (Vardjan et al., 2018; D’Adamo et al., 2021; Figure 1).

Compared with noradrenaline (200μM), which increases cytosolic free d-glucose concentration *via* activation of α_1_-adrenergic receptors and Ca^2+^ signaling due to Ca^2+^-driven extracellular d-glucose uptake (Prebil et al., 2011; Vardjan et al., 2018; D’Adamo et al., 2021; Horvat et al., 2021), L-lactate (20mM) and 3Cl-5OH-BA (0.5mM) decrease cytosolic free d-glucose in astrocytes, as measured with FLII^12^Pglu-700μδ6, a genetically encoded FRET-based glucose nanosensor (Table 1; Vardjan et al., 2018). This is consistent with the fact that extracellular free d-glucose uptake depends primarily on Ca^2+^ signals but not cAMP signals (Horvat et al., 2021), and the fact that extracellular L-lactate and GPR81 agonists trigger increases in cAMP signals but not Ca^2+^ signals in astrocytes (Vardjan et al., 2018; D’Adamo et al., 2021). The observed decrease in cytosolic d-glucose levels in astrocytes exposed to extracellular L-lactate or 3Cl-5OH-BA most likely indicates entry of free d-glucose into the glycolytic pathway.

Thus, L-lactate released from astrocytes may not only activate neurons but may also act in an autocrine manner augmenting its own production in astrocytes. This new positive feedback mechanism of receptor-mediated L-lactate signaling (“metabolic excitability”; Vardjan et al., 2018) that controls astroglial L-lactate production may serve to maintain high intracellular levels of L-lactate, facilitating L-lactate release and thereby generating a concentration gradient between astrocytes and neurons to provide a continuous source of L-lactate to support neural network activity (Mächler et al., 2016; Figure 1). However, because relatively high concentrations of L-lactate (20mM) are needed to facilitate cAMP-mediated aerobic glycolysis (Vardjan et al., 2018; D’Adamo et al., 2021), the new putative astroglial excitatory LLR mechanisms may be particularly relevant under supraphysiologic and pathologic conditions (i.e., ischemia and epilepsy; During et al., 1994; Mosienko et al., 2015), during exercise (Matsui et al., 2017), or at the sites of local extracellular L-lactate increases (L-lactate production in microdomains), if they exist, which needs to be studied in the future (Bergersen and Gjedde, 2012).

## L-Lactate and the Control of Brain Lipid Metabolism

Regulation of brain glucose metabolism has been in the spotlight of the research community for a long time, but the importance of lipid metabolism in brain function has only gained attention in recent years (Panov et al., 2014).

Glial-neuronal coupling of glucose and lipid metabolism was recently suggested to occur as a response to neural activity to protect neurons from lipotoxicity (Liu et al., 2017; Ioannou et al., 2019). The mechanism proposes that L-lactate transport from astrocytes to neurons *via* ANLS triggers *de novo* synthesis of free fatty acids (FFAs) from L-lactate in stressed overstimulated neurons. L-Lactate is decarboxylated in neuronal mitochondria and the resulting acetyl-CoA generates FFAs. Excess of FFAs in neurons is associated with the lipid peroxidation chain reaction and generation of reactive oxygen species (ROS), which may lead to lipotoxicity. To avoid lipotoxicity, excess FFAs are considered to be transferred from neurons to glial cells, particularly astrocytes, in vesicles containing apolipoprotein E-like particles, where they are stored in lipid droplets (LDs; Liu et al., 2015, 2017; Ioannou et al., 2019). FFAs stored in LDs can be used by astrocytes as an energy substrate in β-oxidation (Ioannou et al., 2019), because astrocytes have the capacity to fight mitochondrial overproduction of ROS during β-oxidation. Recently, it was shown that chronic (24h) exposure of both tissue astrocytes and isolated cortical astrocytes in the absence of neurons to 20mM extracellular L-lactate (Smolič et al., 2021) triggers LD accumulation in astrocytes (Table 1). This suggests the existence of an alternative L-lactate-mediated mechanism augmenting LD accumulation in astrocytes. Extracellular L-lactate could affect LD turnover in astrocytes by entering cells *via* MCTs and/or ion channels (Sotelo-Hitschfeld et al., 2015), where L-lactate acts as a substrate for *de novo* FFA synthesis, as shown in oligodendrocytes (Sánchez-Abarca et al., 2001) and neurons (Liu et al., 2017; Ioannou et al., 2019), leading to excess FFA production and FFA storage in LDs to protect astrocytes from lipotoxicity. But extracellular L-lactate may also trigger LD accumulation in astrocytes through actions *via* plasmalemmal LLRs, which needs to be investigated in more detail in the future.

## Conclusion and Perspectives

In conclusion, it is now well established that multiple brain functions are either supported or modulated by L-lactate acting either as a metabolic substrate or signaling molecule. The discovery of signaling properties of L-lactate in astrocytes that are manifested as upregulation in intracellular cAMP production, suggests the existence of a new, as yet unidentified, L-lactate sensitive GPCR coupled to G_s_-proteins in astrocytes. L-Lactate-triggered cAMP signals in astrocytes facilitate aerobic glycolysis with more L-lactate production (metabolic excitability), likely to provide neurons with more L-lactate. Moreover, chronic exposure to L-lactate triggers accumulation of LDs in astrocytes, suggesting that astrocytes switch to lipid metabolism. However, this is achieved only at relatively high extracellular concentrations of L-lactate, implying a role of L-lactate signaling in astrocytes particularly at the sites of putative L-lactate microdomains, under pathologic conditions, or during exercise. Identification of L-lactate-sensitive GPCRs in astrocytes and increasing knowledge on this topic will provide further insights into our understanding of the importance of L-lactate signals in the regulation of brain metabolism and support of brain performance.

## Author Contributions

All authors listed have made a substantial, direct and intellectual contribution to the work, and approved it for publication.

## Funding

The work was supported by grants from the Slovenian Research Agency (P3-0310, J3-6790, J3-9266, and J3-2523) and COST Action CA18133 (ERNEST).

## Conflict of Interest

The authors declare that the research was conducted in the absence of any commercial or financial relationships that could be construed as a potential conflict of interest.

## Publisher’s Note

All claims expressed in this article are solely those of the authors and do not necessarily represent those of their affiliated organizations, or those of the publisher, the editors and the reviewers. Any product that may be evaluated in this article, or claim that may be made by its manufacturer, is not guaranteed or endorsed by the publisher.
